# DOA Estimation for Massive MIMO Systems with Unknown Mutual Coupling Based on Block Sparse Bayesian Learning

**DOI:** 10.3390/s22228634

**Published:** 2022-11-09

**Authors:** Yang Liu, Na Dong, Xiaohui Zhang, Xin Zhao, Yinghui Zhang, Tianshuang Qiu

**Affiliations:** 1 College of Electronic Information Engineering, Inner Mongolia University, Hohhot 010021, China; 2 Faculty of Electronic Information and Electrical Engineering, Dalian University of Technology, Dalian 116024, China

**Keywords:** massive MIMO, DOA estimation, array mutual coupling, sparse bayesian learning (SBL)

## Abstract

Obtaining accurate angle parameters using direction-of-arrival (DOA) estimation algorithms is crucial for acquiring channel state information (CSI) in massive multiple-input multiple-output (MIMO) systems. However, the performance of the existing algorithms deteriorates severely due to mutual coupling between antenna elements in practical engineering. Therefore, for solving the array mutual coupling, the array output signal vector is modeled by mutual coupling coefficients and the DOA estimation problem is transformed into block sparse signal reconstruction and parameter optimization in this paper. Then, a novel sparse Bayesian learning (SBL)-based algorithm is proposed, in which the expectation-maximum (EM) algorithm is used to estimate the unknown parameters iteratively, and the convergence speed of the algorithm is enhanced by utilizing the approximate approximation. Moreover, considering the off-grid error caused by discretization processes, the grid refinement is carried out using the polynomial roots to realize the dynamic update of the grid points, so as to improve the DOA estimation accuracy. Simulation results show that compared with the existing algorithms, the proposed algorithm is more robust to mutual coupling and off-grid error and can obtain better estimation performance.

## 1. Introduction

Massive multiple-input multiple-output (MIMO) technology has been considered one of the most promising technologies for the fifth-generation (5G) and beyond wireless communication networks, because it can obtain a high spatial multiplexing gain and can significantly improve the spatial resolution of array elements [1,2,3]. Accurate angle information obtained through the direction-of-arrival (DOA) estimation plays a crucial role in distinguishing target users from interfering users, reducing pilot pollution, and realizing accurate transmission of effective information in massive MIMO systems [4,5]. Therefore, studies on DOA estimation for massive MIMO systems have important research value and application significance in the field of wireless communications [6,7].

Traditional subspace-based DOA estimation algorithms, such as multiple signal classification (MUSIC) and estimation of signal parameters via rotational invariance techniques (ESPRIT), have been proven to provide high-precision angle estimation, but their computational complexity is relatively high in massive MIMO systems [8,9]. In view of that, numerous improved algorithms have been developed based on these algorithms in recent decades [10,11]. However, as the electromagnetic environment becomes increasingly complex and the direction-finding scenario worsens, these algorithms cause wastage of resources and the severe deterioration of direction-finding performance under low signal-to-noise ratio (SNR), small snapshots, and even large array errors. In addition, these algorithms cannot completely deal with the above-mentioned problems due to the limitation of the subspace framework.

Compressed sensing technology has good applicability and can accurately recover the original signal from low-dimension measurement data using non-zero coefficients [12]. It should be noted that, in compressed sensing technology, the sampling rate is not related to the highest frequency, and a sampling method depends on the sparsity and structure information of a signal. Recently, it has been proposed to use Bayesian compressed sensing [13] to address problems in DOA estimation. Compared with greedy and convex relaxation algorithms, sparse Bayesian learning (SBL) does not require strict information about the number of sources and can accurately reconstruct sparse signals. In view of the two major problems (i.e., small array aperture and large modeling error) existing in the off-grid SBL DOA estimation methods for nested arrays, [14] provided a novel grid refinement method based on a new data formulation to eliminate the modeling error caused by off-grid gaps. In [15,16,17], the authors developed Bayesian learning-based strategies for solving the DOA estimation problem. In [15], it has been proven that SBL can perform remarkably better than traditional beamforming and MUSIC. Since the most frequently used real-valued transformation is suitable for uniform linear arrays only, an innovative real-valued transformation with a lower computational burden was developed in [16], using the virtual steering of linear arrays. In addition, two efficient SBL-based methods for DOA estimation with arbitrary linear arrays were proposed. To diagnose the blocked elements timely under a low SNR, [17] developed an adaptive iterative diagnostic approach based on clustering block SBL, which has a high estimation accuracy in solving the coupling of DOA and antenna blockage estimation.

In practical massive MIMO systems, the performance of conventional DOA estimation algorithms degrades severely or even fails completely due to the mutual coupling between antenna elements [18,19]. The DOA estimation problem for MIMO radar systems with unknown mutual coupling was transformed into a sparse reconstruction problem, in which the off-grid effect and mutual coupling effect are considered simultaneously [20]. Then, a novel sparse Bayesian learning with the mutual coupling (SBLMC) method was proposed. To this end, a ε-negative meta-surface superstrate and *I*-shaped resonators were introduced to reduce mutual coupling [21,22]. Considering that the spatial covariance matrix (SCM), which plays an important role in DOA estimation, cannot be obtained in hybrid massive MIMO systems because the received signals cannot reach the receiver directly, the SCM reconstruction was performed using different algorithms [23,24]. Since random sampling based two-dimensional (2D) DOA estimation in massive MIMO systems can lead to matrix completion, an efficient matrix completion method based on the alternating direction method of multipliers was proposed in [25] to reduce power consumption. In [26], a new higher-order propagator method (HOPM) was designed to estimate the tensor-based signal subspace for 2D DOA estimation, which can achieve high estimation accuracy at low computational complexity. Moreover, a higher-order unitary PM (HOUPM) was proposed to improve the performance further. In [27], a deep-learning-based DOA estimation algorithm was proposed for hybrid massive MIMO systems with a uniform circular array (UCA). This system can realize comparable and, in certain cases, even superior performance at lower computational complexity compared with the maximum likelihood (ML) method. For the 2D DOA and polarization estimation of non-circular signals in polarized massive MIMO systems with uniform rectangular arrays (URAs), an efficient quaternion non-circular MUSIC (QNC-MUSIC) algorithm, which has a high estimation accuracy but low computational complexity, especially for a large source number was proposed in [28]. In [29], a spatial spectrum fusion estimation and localization (SSFEAL) algorithm for massive MIMO systems with UCAs, which can obtain decimeter-level localization accuracy and meet the requirement of future networks, was presented. To solve the two main challenges in Terahertz dynamic array-of-subarray (DAoAS) systems, [30] developed a DAoAS-MUSIC algorithm and a deep convolutional neural network (DCNN)-based algorithm for DOA estimation. Both proposed algorithms can realize super-resolution DOA estimation, and the DCNN-based algorithm can perform better than the DAoAS-MUSIC at a high SNR. In [31], an efficient method for performing the 2D DOA estimation of incoherently distributed sources with gain-phase perturbations was proposed. The proposed method can improve the estimation accuracy efficiently and also is unaffected by the array gain phase disturbance. To overcome the challenges brought by unknown mutual coupling and small samples in accurate DOA estimation for massive MIMO systems, an improved DOA estimation method based on eigenvalue comparison and real-valued MUSIC was proposed in [32]. This method can improve both the performance and the computational efficiency of DOA estimation. It is worth noting that although there have been many studies on DOA estimation algorithms for massive MIMO systems, most of them have ignored the impact of mutual coupling error on DOA estimation performance and have only slightly considered the conditions of low SNR and a small number of snapshots.

To address the shortcomings of the existing work, this paper develops an innovative algorithm based on the block SBL (BSBL) to solve the DOA estimation for massive MIMO systems with unknown array mutual coupling under the conditions of low SNR and small snapshots. Considering the existence of array errors in the actual environment, the array defects are addressed, and the DOA estimation problem is transformed into a block sparse signal reconstruction and parameter optimization problem. Then, structural information of a signal is used, and the cost function of the proposed algorithm is optimized, thus significantly improving the performance of the sparse reconstruction algorithm. The proposed algorithm is verified by simulations, and simulation results demonstrate the excellent performance of the proposed algorithm. The main contributions of this paper can be summarized as follows:

1. Considering the array mutual coupling error, which can severely deteriorate the DOA estimation performance, an array output vector is constructed based on the block sparsity of the original signal, where the array flow matrix of the signal is improved by the mutual coupling coefficient. Therefore, the DOA estimation problem is regarded as a block sparse signal reconstruction and parameter optimization problem.
2. The expectation-maximization (EM) algorithm is used to estimate hyperparameters and noise parameters to reconstruct the sparse signal. To enhance algorithm convergence speed, the boundary of the cost function is obtained by adopting the approximate approximation, and the hyperparameter representing the signal correlation is optimized, which improves the convergence speed to a large extent.
3. Considering the high computational complexity and low estimation accuracy of the equal-spacing angle space division, this study refines the angle space grids by finding the roots of the polynomial to realize the dynamic update of grid points in a discrete space; then a spatial screening of the updated grid is performed to achieve DOA estimation. In addition, the threshold is set to determine the grid points to be updated, thus improving the estimation efficiency. After the updating process, the grid points are closer to the real DOAs, which efficiently eliminates the off-grid error and improves the estimation accuracy. Moreover, an improved SBL-based algorithm (ImBSBL) is proposed for DOA estimation. Simulation results illustrate that compared with the existing algorithms, the proposed algorithm is more robust to mutual coupling and the off-grid error and can obtain better estimation performance.

The rest of this paper is organized as follows. In Section 2, the signal model is introduced. In Section 3, a DOA estimation algorithm for massive MIMO systems based on addressing unknown mutual coupling under a low SNR and small snapshots is introduced. The simulation results are given in Section 4. Finally, the conclusions are drawn in Section 5.

## 2. System Model

Consider a massive MIMO system with a uniform linear array (ULA) consisting of *M* antenna elements, receiving *K* far-field narrowband signals from distinct directions $\theta_{k}$(k=1,2,⋯,K). From the perspective of spatial sparsity, the whole space can be treated as an angle set ${\left\{{\hat{\theta }}_{i}\right\}}_{i=1}^{N}$ that evenly divides the DOA range $\left[-\frac{\pi }{2},\frac{\pi }{2}\right]$ into *N* segments, N(N≫K), where ${\hat{\theta }}_{i}$ denotes the possible angle of the incident signal. When the incident signal approaches the array from angle $\theta_{k}={\hat{\theta }}_{i}$, the spatial energy at that angle accumulates, forming a significant peak point, while the spatial energy at all other angles is zero. Thus, the array output vector can be expressed by:

(1) $$y\left(t\right)=\bar{A}s\left(t\right)+n\left(t\right),$$

where y(t) and n(t) are the M×1 observation matrix and observation noise matrix, respectively; s(t) is an N×1 sparse target signal.

This makes it the best represented in the span of the base matrix, $\bar{A}\in C^{M\times N}$ is the estimation problem to solve. To reconstruct the target signal accurately, the base matrix $\bar{A}$ is required to be an overcomplete matrix (i.e., N>M), and it is defined as follows:

(2) $$\bar{A}=\left[a\left({\hat{\theta }}_{1}\right),\ldots ,a\left({\hat{\theta }}_{N}\right)\right],$$

where $a\left({\hat{\theta }}_{i}\right)={\left[1,e^{-j2\pi \frac{d}{\lambda }\sin{\hat{\theta }}_{i}},\ldots ,e^{-j2\pi \frac{d}{\lambda }\left(M-1\right)\sin{\hat{\theta }}_{i}}\right]}^{T}$ and ${\left[\cdot \right]}^{T}$ represents transposition.

Since a unique solution cannot be obtained when s(t) is solved according to Equation (1), it is necessary to constrain the signal part of measurement data. In the presence of noise n(t) with zero mean and variance $\sigma^{2}$, the DOA estimation problem can be regarded as a $\ell_{0}$-norm optimization problem, which is defined by:

(3) $$\hat{s}\left(t\right)=\arg\min_{s}{∥s∥}_{0}\;\;s.t.\;\;∥y-\bar{A}s∥\leq \sigma^{2}.$$

## 3. Improved BSBL-Based DOA Estimation Algorithm for Massive MIMO System with Unknown Mutual Coupling

### 3.1. Mutual Coupling Modeling

Mutual coupling error is common in applications using antenna arrays, and it typically occurs between adjacent elements, particularly when a system operates at a high frequency [16]. The mutual coupling matrix of a ULA can be modeled as a symmetric Toeplitz matrix, which is given by:

(4) $$C=\left[\begin{array}{cccccc} c_{1} & c_{2} & \cdots & c_{L} & \cdots & c_{M}\\ c_{2} & c_{1} & c_{2} & \; & \ddots & \vdots \\ \vdots & c_{2} & c_{1} & c_{2} & \; & c_{L}\\ c_{L} & \; & c_{2} & \ddots & \ddots & \vdots \\ \vdots & \ddots & \; & \ddots & c_{1} & c_{2}\\ c_{M} & \cdots & c_{L} & \cdots & c_{2} & c_{1} \end{array}\right],$$

where $c_{i}\left(i=1,2,\ldots ,M\right)$ denotes the mutual coupling coefficient that satisfies the condition of $1=c_{1}>\cdots >\left|c_{L}\right|>\left|c_{L+1}\right|=\cdots =\left|c_{M}\right|=0$; *L* is the degree of freedom of the mutual coupling matrix, which is unknown in practice. Considering the influence of mutual coupling, Equation (1) can be modified as follows:

(5) $$y\left(t\right)=C\bar{A}\left(t\right)+n\left(t\right).$$

Furthermore, to decompose the angle information and mutual coupling coefficient of signal Equation (5), according to the theorem [33], the following equation can be derived:

(6) Ca=Q(a)c,

where C is a mutual coupling matrix, and $C=\;\mathrm{Toeplitz}\;\left\{c\right\}\in C^{M\times M}$, where c is the vector consisting of the first-row elements of C; a is a complex vector, $a\in C^{M\times 1}$, which represents the steering vector.

To solve an M×L transformation matrix Q(a) that can realize the decoupling of position factor and mutual coupling factor, C is decomposeed into C=WZ, where $W=\left[W_{1},..,W_{L}\right]$ and Z=c⊗I denote M×ML and ML×M matrices, respectively; I represents the identity matrix; ⊗ stands for the Kronecker product.

The (i,j)-th element in the *l*-th subblock of W can be calculated by:

(7) $${\left[W_{l}\right]}_{ij}=\left\{\begin{array}{c} 1,\;\mathrm{if}\;{\left[C\right]}_{ij}=c_{l}\\ 0,\;\mathrm{otherwise}\; \end{array}\right.,$$

Therefore, Equation (6) can be rewritten as follows:

(8) $$\mathrm{Ca}=Wc⊗a=W\left[\begin{array}{c} c_{1}a\\ c_{2}a\\ \vdots \\ c_{L}a \end{array}\right]=\left[W_{1}a,W_{2}a,\ldots ,W_{L}a\right]\left[\begin{array}{c} c_{1}\\ c_{2}\\ \vdots \\ c_{L} \end{array}\right].$$

According to Equation (6) and Equation (8), Q(a) is a function of the incident signal with $\hat{\theta }$, so it can be expressed as $Q(\hat{\theta })$, and then it holds that $Q\left(\hat{\theta }\right)=\left[W_{1}a,W_{2}a,\ldots ,W_{L}a\right]$. Thus, Equation (5) can be rewritten as follows:

(9) $$\begin{array}{cc} y(t) & =C\left[a\left({\hat{\theta }}_{1}\right),\ldots ,a\left({\hat{\theta }}_{N}\right)\right]s\left(t\right)+n\left(t\right)\\ \; & =\left[Q\left({\hat{\theta }}_{1}\right)c,\ldots ,Q\left({\hat{\theta }}_{N}\right)c\right]s\left(t\right)+n\left(t\right)\\ \; & =D\left(\hat{\theta }\right)x\left(t\right)+n\left(t\right) \end{array},$$

where $D\left(\hat{\theta }\right)=\left[Q\left({\hat{\theta }}_{1}\right),\ldots ,Q\left({\hat{\theta }}_{N}\right)\right]$ is an array flow pattern matrix considering the mutual coupling factor, and the received signal of the array is given by:

(10) $$x\left(t\right)=s\left(t\right)⊗c=\left[\begin{array}{c} s_{1}\left(t\right)c\\ s_{2}\left(t\right)c\\ \vdots \\ s_{N}\left(t\right)c \end{array}\right].$$

According to Equation (10), $x\left(t\right)={\left[x_{1}^{T}\left(t\right),\ldots ,x_{N}^{T}\left(t\right)\right]}^{T}$ is the signal with a block structure. In addition, since each row in s(t) corresponds to a signal in a potential incident direction and the element in a row is non-zero when this direction is the real DOA direction, s(t) indicates the row is sparse. Therefore, this paper regards the DOA estimation problem as a problem of block sparse signal reconstruction and parameter optimization.

### 3.2. Sparse Bayesian Solution

Since block sparsity can reduce the search degree of freedom in the signal space and can improve the reconstruction accuracy and robustness of an algorithm [34], sparse reconstruction algorithms can accurately reconstruct sparse signals. In view of this, this work employs the EM algorithm to reconstruct the sparse signal and realize the DOA estimation for the array output signal model, as shown in Equation (9). For convenience, Equation (9) can be briefly rewritten as follows:

(11) y=Dx+n,

where x denotes the block sparse signal.

To avoid over-matching, an SBL method can fully mine and use the prior information on data and constrain the estimated signals, which not only makes the signal more easily meet the sparse condition but also contributes to the high-precision recovery of a sparse reconstruction algorithm [35]. Assuming that the blocks are not correlated with each other; then the block signal $x={\left[x_{1}^{T},\ldots x_{g}^{T}\right]}^{T}$ can be modeled as follows:

(12) $$p\left(x|\gamma_{i},B_{i}\right)=CN\left(x;0,\Gamma \right),$$

where *CN* denotes the complex Gaussian distribution, $\gamma_{i}$ controls the sparsity of a block, $B_{i}$ captures the intra-block correlation structure [36], and $\Gamma ={\mathrm{diag}}^{-1}\left(\gamma_{1}B_{1},\ldots ,\gamma_{g}B_{g}\right)$. The observation noise vector n is assumed to have the distribution of $p\left(n\right)∼CN\left(0,\beta^{-1}I\right)$, where β represents the noise variance. Using the block model Equation (11), the Gaussian likelihood function with mean Dx and variance $\beta^{-1}$ can be obtained as follows:

(13) $$p\left(y|x;\beta \right)=CN\left(y;Dx,\beta^{-1}I\right),$$

where y is the observation vector, $y\in C^{M\times 1}$, and it obeys the Gaussian distribution.

The posterior probability can be obtained by Equations (12) and (13) using Gauss identity property as follows:

(14) $$\begin{array}{cc} \; & p\left(x|y;\left\{\gamma_{i},B_{i}\right\},\beta \right)\cdot p\left(y|\left\{\gamma_{i},B_{i}\right\},\beta \right)\\ = & p\left(y|x,\beta \right)p\left(x|\left\{\gamma_{i},B_{i}\right\}\right)\\ = & CN\left(y|Dx,\beta^{-1}I\right)CN\left(x|0,\Gamma \right)\\ = & CN(x|\mu ,\Sigma )CN(y|0,T) \end{array},$$

where

(15) $$T=\beta^{-1}I+D\Gamma D^{H},$$

(16) $$\Sigma^{-1}=\Gamma^{-1}+D^{H}\beta D,$$

(17) $$\mu =\Sigma D^{H}\beta y$$

where ${\left[\cdot \right]}^{H}$ represents conjugate transpose.

However, there will occur a large numerical error in calculating $\Gamma^{-1}$ when $\gamma_{i}$ is too small or close to zero; thus, Equation (16) is modified as follows:

(18) $$\Sigma =\Gamma -\Gamma D^{H}T^{-1}D\Gamma .$$

Furthermore, to make the calculation of μ not related to the update of Σ, μ is simplified as follows:

(19) $$\begin{array}{cc} \mu & =\left(\beta^{-1}\Gamma^{-1}+D^{H}D\right)D^{H}y\\ \; & =D^{H}{\left(\beta^{-1}\Gamma^{-1}+DD^{H}\right)}^{-1}y\\ \; & =D^{H}\Gamma T^{-1}y \end{array},$$

where μ and Σ denote the mean and covariance, respectively, and they are estimated by the EM method.

To find a sparse solution, the global minimum of the cost function [37] is calculated by:

(20) $$\begin{array}{cc} L\left(\left\{\gamma_{i},B_{i}\right\},\beta \right) & =-2\log p\left(y|\left(\left\{\gamma_{i},B_{i}\right\},\beta \right)\right)\\ \; & =\log|T|+y^{H}T^{-1}y \end{array}.$$

Next, using the Sherman–Morrison matrix identity formula,

(21) $${\left(A+\mathrm{XB}X^{T}\right)}^{-1}=A^{-1}-A^{-1}X{\left(B^{-1}+X^{T}A^{-1}X\right)}^{-1}X^{T}A^{-1},$$

(22) $$\left|A+XBX^{T}\right|=\left|B∥A\right|\left|B^{-1}+X^{T}A^{-1}X\right|,$$

where A and B are invertible matrices, Equation (20) can be written as follows:

(23) $$L=\left(\sum_{i=1}^{g}\log\left|\gamma_{i}B_{i}\right|-N\log\left|\beta \right|+\log\left|\Sigma^{-1}\right|\right)+\left({\beta ∥y-D\mu ∥}_{2}^{2}+\mu^{H}\Gamma^{-1}\mu \right).$$

Then, the solution to paraments $\gamma_{i}$, $B_{i}$, and β is obtained. First, the partial derivative of Equation (23) is given by:

(24) $$\frac{\partial L}{\partial \gamma_{i}}=\frac{d_{i}}{\gamma_{i}}-\mathrm{Tr}\left[\frac{B_{i}^{-1}\left(\mu_{i}\mu_{i}^{H}+\Sigma_{i}\right)}{\gamma_{i}^{2}}\right],$$

(25) $$\frac{\partial L}{\partial B_{i}}=B_{i}^{-1}-\gamma_{i}^{-1}B_{i}^{-1}\left[\Sigma_{i}+\mu_{i}\mu_{i}^{H}\right]B_{i}^{-1},$$

(26) $$\frac{\partial L}{\partial \beta }=-\frac{N}{\beta }+\mathrm{Tr}\left[\Sigma D^{H}D\right]+{∥y-D\mu ∥}_{2}^{2},$$

where Tr[•] represents trace, $\mu_{i}$ denotes the *i*-th row of μ, and $\Sigma_{i}$ denotes the *i*-th main diagonal element of Σ.

Assume that values of Equations (24)–(26) are zeros; then, it can be written as follows:

(27) $$\gamma_{i}=\frac{1}{d_{i}}\mathrm{Tr}\left[B_{i}^{-1}\left(\Sigma_{i}+\mu_{i}\mu_{i}^{H}\right)\right],$$

(28) $$B_{i}=\frac{\left[\Sigma_{i}+\mu_{i}\mu_{i}^{H}\right]}{\gamma_{i}},$$

(29) $$\beta =\frac{N}{{∥y-D\mu ∥}^{2}+\mathrm{Tr}\left[\Sigma D^{H}D\right]},$$

where $\gamma_{i}$ and β the parameters that affect the convergence speed of the SBL algorithm.

Compared with the influence of parameter β on algorithm performance, parameter $\gamma_{i}$ has a greater influence on algorithm performance; namely, different update approaches of $\gamma_{i}$ affect algorithm performance, altering its convergence speed performance [18]. Moreover, the updated β and $B_{i}$ both affect the reconstruction performance of the sparse recovery algorithm, but the effect of β is more significant. If optimal β cannot be obtained, the reconstruction and recovery performances of an algorithm cannot be improved even if other parameters are optimized. A number of studies have shown that the parameter β in an EM algorithm has poor robustness under a certain SNR because the non-diagonal elements in block elements interfere with the elements on the main diagonal [35]. Parameter β is obtained by

(30) $$\beta =\frac{N}{{∥y-D\mu ∥}_{2}^{2}+\sum_{i=1}^{g}\mathrm{Tr}\left[\Sigma_{i}D_{i}^{H}D_{i}\right]}.$$

It should be noted that $B_{i}$ affects only the local convergence performance, and the EM algorithm specifies different constraints for different blocks, which can lead to the overfitting problem. Therefore, according to the parameter average idea, so $B_{i}=B$, and it holds that:

(31) $$B=\frac{1}{g}\sum_{i=1}^{g}\frac{\Sigma_{i}+\mu_{i}{\left(\mu_{i}\right)}^{H}}{\gamma_{i}}.$$

Since $B_{i}=cc^{H}$ contains the mutual coupling information under unknown mutual coupling, the mutual coupling coefficient can be estimated using the first column of B, and the mutual coupling matrix can be obtained. Finally, when the parameters β and ${\left\{\gamma_{i},B_{i}\right\}}_{i}$ are estimated, the Maximum-A-Posteriori (MAP) estimate of x, which is denoted by $\hat{x}$, can be obtained by:

(32) $$\hat{x}\leftarrow \Gamma D^{H}{\left(\beta^{-1}I+D\Gamma D^{H}\right)}^{-1}y.$$

### 3.3. Optimized Hyperparameter Updating

In the previous subsection, a sparse Bayesian solution is described in detail, and it is explained that the EM algorithm is applied to obtain the updated parameters and sparse recovery result of a signal. However, convergence speed affects algorithm efficiency significantly. Particularly, in massive MIMO, enhancing the convergence speed can effectively reduce algorithm complexity. Numerous studies have shown that parameter $\gamma_{i}$ affects the convergence speed of an EM algorithm [38,39]. Therefore, this section optimizes parameter $\gamma_{i}$ and then refines the grid by finding roots of polynomials. In view of this, this study proposes an improved ImBSBL algorithm for DOA estimation, whose main aim is to find an alternative method through approximate approximation and obtain the boundary of the cost function.

The following analysis will be conducted based on Equation (20), whose first term is concave while the second term is convex. First, the upper bound of the first term is obtained by:

(33) $$\begin{array}{cc} \log\left|\beta^{-1}I+D\Gamma D^{H}\right| & \leq \log\left|\beta^{-1}I+D\Gamma^{*}D^{H}\right|\\ \; & +\sum_{i=1}^{g}\mathrm{Tr}\left(T^{*-1}D_{i}B_{i}D_{i}^{H}\right)\left(\gamma_{i}-\gamma_{i}^{*}\right)\\ \; & =\sum_{i=1}^{g}\mathrm{Tr}\left(T^{*-1}D_{i}B_{i}D_{i}^{H}\right)\gamma_{i}\\ \; & +\log\left|T^{*}\right|-\sum_{i=1}^{g}\mathrm{Tr}\left(T^{*-1}D_{i}B_{i}D_{i}^{H}\right)\gamma_{i}^{*} \end{array}.$$

where ${\left[\cdot \right]}^{*}$ represents conjugation.

Then, by substituting Equation (33) into Equation (20), the following inequality is obtained:

(34) $$\begin{array}{cc} L(\gamma ) & \leq \sum_{i=1}^{g}\mathrm{Tr}\left(T^{*-1}D_{i}B_{i}D_{i}^{H}\right)\gamma_{i}+\log\left|T^{*}\right|\\ \; & -\sum_{i=1}^{g}\mathrm{Tr}\left(T^{*-1}D_{i}B_{i}D_{i}^{H}\right)\gamma_{i}^{*}\\ \; & +y^{H}{\left(\beta I+D\Gamma D^{H}\right)}^{-1}y\\ \; & =\tilde{L}\left(\gamma \right) \end{array}.$$

The abovementioned inequality indicates that $\tilde{L}\left(\gamma \right)$ is a convex function, so its minimum can be obtained at the minimum of the variable γ and it should satisfy the condition of $L\left(\gamma_{\min}\right)\leq \tilde{L}\left(\gamma_{\min}\right)\leq \tilde{L}\left(\gamma^{*}\right)=L\left(\gamma^{*}\right)$. Thus, minimizing the right side of Equation (34) will reduce the maximum value of the cost function.

Furthermore, the second term of Equation (20) is given by:

(35) $$y^{H}{\left(\beta^{-1}I+D\Gamma D^{T}\right)}^{-1}y=\min_{x}\left[\frac{1}{\beta }{∥y-Dx∥}_{2}^{2}+x^{H}\Gamma^{-1}x\right].$$

Thus, the substitution function is defined as follows:

(36) $$\begin{array}{cc} \tilde{L}\left(\gamma \right) & =\sum_{i=1}^{g}\mathrm{Tr}\left(T^{*-1}D_{i}B_{i}D_{i}^{H}\right)\gamma_{i}+\log\left|T^{*}\right|\\ \; & -\sum_{i=1}^{g}\mathrm{Tr}\left(T^{*-1}D_{i}B_{i}D_{i}^{H}\right)\gamma_{i}^{*}\\ \; & +\min_{x}\frac{1}{\beta }{∥y-\Phi x∥}_{2}^{2}+x^{H}\Gamma^{-1}x \end{array}.$$

Next, let $\frac{\partial \tilde{L}\left(\gamma \right)}{\partial \gamma_{i}}=0$; then, the update of $\gamma_{i}$ is given by:

(37) $$\gamma_{i}\leftarrow \sqrt{\frac{\mu_{i}^{H}B_{i}^{-1}\mu_{i}}{\mathrm{Tr}\left[D_{i}^{H}T^{-1}D_{i}B_{i}\right]}}.$$

Since the sparse model is divided based on the grid, when the incident angle of a signal is not in the divided grid, there will be an off-grid error in the DOA estimation result. To reduce the influence of the off-grid error on the estimation result, this paper refines the gird using the approach of finding roots of polynomials. To obtain the grid parameter ${\hat{\theta }}_{i}\left(i=1,\ldots ,N\right)$, logarithmic terms irrelevant to formula derivation are ignored, and the following equation is maximized:

(38) $$\begin{array}{c} E_{p\left(x|y;\left\{\gamma_{i},B_{i}\right\},\beta \right)}\left[\log p\left(y|x;\beta \right)\right]\\ =-\beta E_{p\left(x|y;\left\{\gamma_{i},B_{i}\right\},\beta \right)}\left[{∥y-Dx∥}_{2}^{2}\right]\\ ={-\beta ∥y-D\mu ∥}_{2}^{2}-\beta \mathrm{Tr}\left[D\Sigma D^{H}\right] \end{array}.$$

Then, to refine ${\hat{\theta }}_{i}$, let the partial derivative of Equation (38) with respect to the steering vector $V_{{\hat{\theta }}_{i}}=e^{-j2\pi d/\lambda \sin{\hat{\theta }}_{i}}$ be zero, which can be expressed by:

(39) $$\frac{{\partial ∥y-D\mu ∥}_{2}^{2}}{\partial V_{{\hat{\theta }}_{i}}}+\frac{\partial \mathrm{Tr}\left[D\Sigma D^{H}\right]}{\partial V_{{\hat{\theta }}_{i}}}=0.$$

According to the idea of a polynomial root, Equation (39) can be expressed in the form of a polynomial with respect to ${\hat{\theta }}_{i}$ using algebraic manipulation. Since Equation (39) contains the first derivative of the direction vector with respect to $V_{{\hat{\theta }}_{i}}$, its polynomial form has M−1 roots in the complex field, and roots of the refined grid parameters should have unit amplitude. However, the roots cannot fall in the unit circle exactly due to the noise in a real environment. Assuming that $V_{{\hat{\theta }}_{i}^{*}}$ denotes the root closest to the unit circle, then, it can be written as:

(40) $${\hat{\theta }}_{i^{*}}^{\mathrm{new}\;}=\arcsin\left(-\frac{\lambda }{2\pi d}\cdot \mathrm{angle}\left(V_{{\hat{\theta }}_{i}^{*}}\right)\right).$$

Thus, the parameters for refining the grid are obtained. Considering that grid refinement for real DOA, which is close to or equal to the initial grid point, will increase the estimation error of the algorithm, a threshold should be defined to judge whether to perform grid refinement. The initial grid needs to be refined by ${\hat{\theta }}_{i^{*}}^{new}$ when ${\hat{\theta }}_{i^{*}}^{\mathrm{new}}\in \left[\frac{{\hat{\theta }}_{i^{*}-1}+{\hat{\theta }}_{i^{*}}}{2},\frac{{\hat{\theta }}_{i^{*}}+{\hat{\theta }}_{i^{*}+1}}{2}\right]$; otherwise, the refinement is not necessary. Since it is not effective to refine all grid points in each iteration, a threshold η is set, and the Frobenius norm is used to select the first η(1≤η≤M) largest elements of μ and obtain their indexes, which are used to determine the grid to be updated [40]. In this study, the number of sources is set to η=M.

For clarity, the proposed ImBSBL algorithm for DOA estimation with unknown mutual coupling is summarized in Algorithm 1. It is worth noting that this research does not involve the synchronization of complex dynamic networks. Different from [14], our work not only takes the off-grid factor into account, but also deals with the influence of array mutual coupling on DOA estimation. In addition, compared with [20], the proposed algorithm improves the convergence speed of the EM algorithm when estimating parameters by approximate approximation.
**Algorithm 1:** The Proposed ImBSBL Algorithm for DOA Estimation with Unknown Mutual Coupling and Off-grid Error.1:**Input:** Received signal x;2:**Initialization:** Initialize the number of iterations κ and parameters $\gamma^{\left(\kappa \right)}$, $B^{\left(\kappa \right)}$, and $\beta^{\left(\kappa \right)}$;3:**while** not converged **do**4:   Calculate $\mu^{\left(\kappa +1\right)}$ and $\Sigma^{\left(\kappa +1\right)}$ using Equation (19) and Equation (18), respectively.5:   Update $\gamma^{\left(\kappa +1\right)}$, $B^{\left(\kappa +1\right)}$, $\beta^{\left(\kappa +1\right)}$ according to Equation (37), Equation (31) and Equation (30), respectively;6:   Update ${\hat{\theta }}_{i^{*}}^{\left(\kappa +1\right)}$ by Equation (40);7:   **if** $∥\gamma^{\left(\kappa +1\right)}-\gamma^{\left(k\right)}∥/∥\gamma^{\left(k\right)}∥<10^{-4}$ or κ=1000 **then**8:     break9:   **else**10:     Set κ=κ+1;11:     Return to Step 4.12:   **end if**13:**end while**14:**Output:**$\mu^{\left(\kappa +1\right)}$ and ${\hat{\theta }}_{i^{*}}^{\left(\kappa +1\right)}$.15:Perform one dimensional spectrum search on new ${\hat{\theta }}_{i^{*}}^{\left(\kappa +1\right)}$ to find the *K* maximum values to perform the DOA estimation.

## 4. Simulation Results

Several experiments were performed to illustrate the superiority of the proposed algorithm over the existing algorithms, including the root SBL (ROSBL) algorithm based on root finding [40], the classical high-resolution high-precision MUSIC algorithm [41], the multi-resolution focal underdetermined system solver (MFOCUSS) algorithm [42] in the convex optimization algorithm, and the sparse Bayesian array calibration (SBAC) algorithm based on the signal observation error model [43].

### 4.1. Effect of Mutual Coupling on DOA Estimation

**Figure 1:**
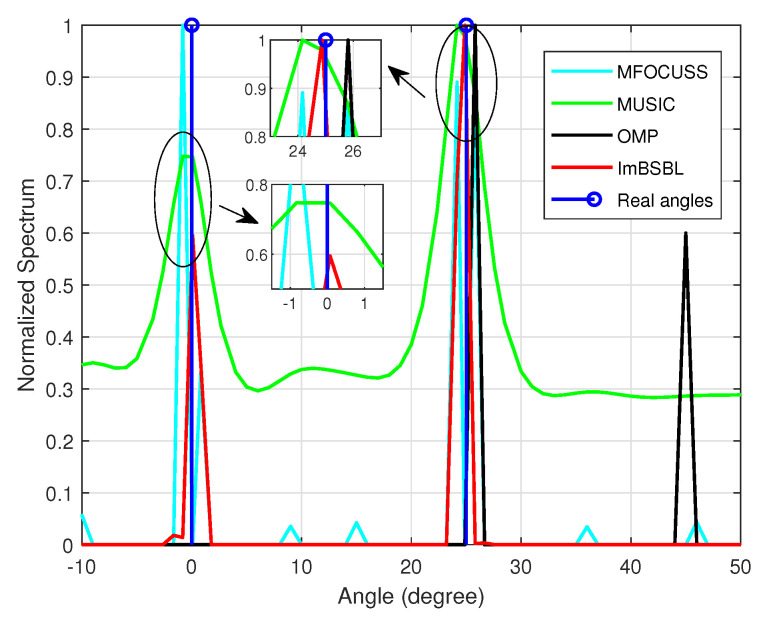
The impact of array imperfections on the spatial spectra.

The mutual coupling relationship between the elements was described by [44]

(41) $$c_{n}=\left\{\begin{array}{c} \left(1+\xi \right)e^{j\phi }10\frac{\alpha_{c}\left(1+0.5n\right)}{20},n<5\\ 0,\;\mathrm{otherwise}\; \end{array}\right.,$$

where ξ∼u([−0.05,0.05]), φ∼u([0,2π]) and $\alpha_{c}$ are used to measure the mutual coupling effect between adjacent elements. A ULA with M=16 elements is used to receive K=2 far-field signals arriving at the incident angles of $\theta =0^{\circ }$ and $\theta =25^{\circ }$. The SNR is set to 5 dB, the number of snapshots is set to 100, and the grid interval is $1^{\circ }$. The spatial spectrum of the MFOCUSS, MUSIC, OMP, and ImBSBL algorithms are presented in Figure 1, where it can be observed that the proposed ImBSBL algorithm has two sharp spectral peaks, which are close to the actual incident positions. Compared with the OMP algorithm, the MFOCUSS algorithm is more accurate but produces many smaller spectral peaks. The MUSIC algorithm performs the worst among all algorithms, having no sharp spectral peak, and the peak is far away from the real position. The reason for these results is that, among the compared algorithms, only the proposed ImBSBL algorithm considers the mutual coupling factor and off−grid error, which could severely deteriorate the DOA estimation performance. Therefore, its estimation performance is optimal.

**Figure 2:**
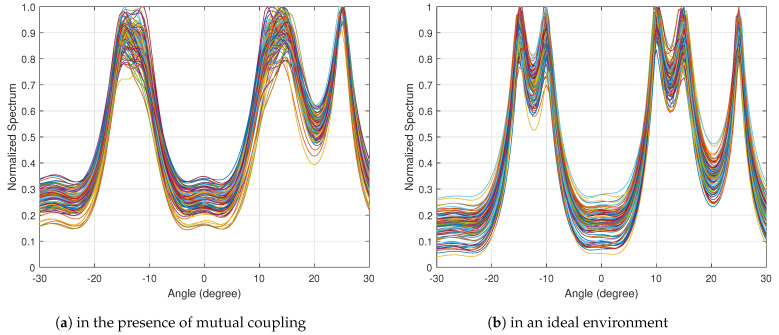
The spatial spectra of the MUSIC algorithm.

**Figure 3:**
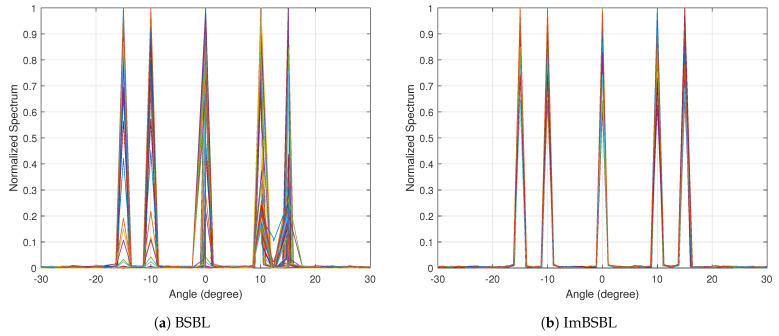
The spatial spectra of the BSBL algorithm and the proposed ImBSBL algorithm in an ideal environment.

**Figure 4:**
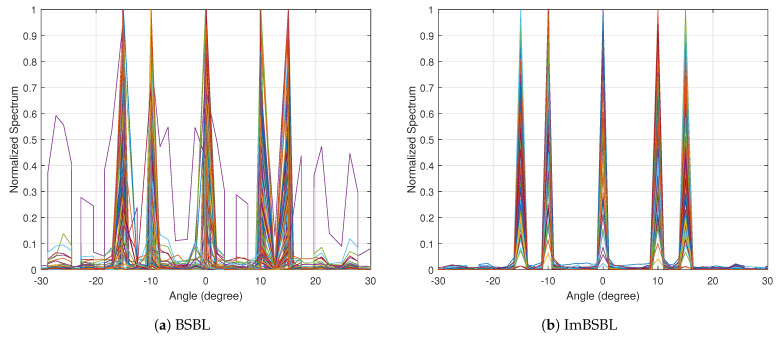
The spatial spectra of the BSBL algorithm and the proposed ImBSBL algorithm in the presence of mutual coupling.

The incident angle are set to different values, including $\theta =-15^{\circ }$, $\theta =-10^{\circ }$, $\theta =0^{\circ }$, $\theta =10^{\circ }$ and $\theta =15^{\circ }$, while the other parameters are set as defined above. The DOA estimation spatial spectrum of the MUSIC algorithm in the presence of mutual coupling and in an ideal environment obtained by 100 Monte−Carlo runs is presented in Figure 2. The performances of the BSBL algorithm and the proposed ImBSBL algorithm in an ideal environment and in the case of mutual coupling are presented in Figure 3 and Figure 4, respectively. As shown in Figure 2, the conventional MUSIC algorithm could accurately estimate the real incident angles of signals from multiple sources in an ideal environment. However, the mutual coupling has a destructive effect and severely affects the resolution and estimation accuracy of the MUSIC algorithm. Furthermore, as presented in Figure 3, although the BSBL algorithm could accurately estimate the real incident angles of signals in the ideal environment, its performance significantly degrades in the presence of mutual coupling. Compared with the BSBL algorithm, the proposed ImBSBL algorithm is more robust to mutual coupling, and could accurately estimate the true incident angles of signals from multiple sources with a lower side lobe and smaller power leakage without pseudo peaks. This is because the mutual coupling factors are decoupled by utilizing the symmetric Toeplitz property and the banded structure of the mutual coupling matrix. Furthermore, the dynamic update of grid points by exploiting the polynomial roots can also improve the DOA estimation precision.

**Figure 5:**
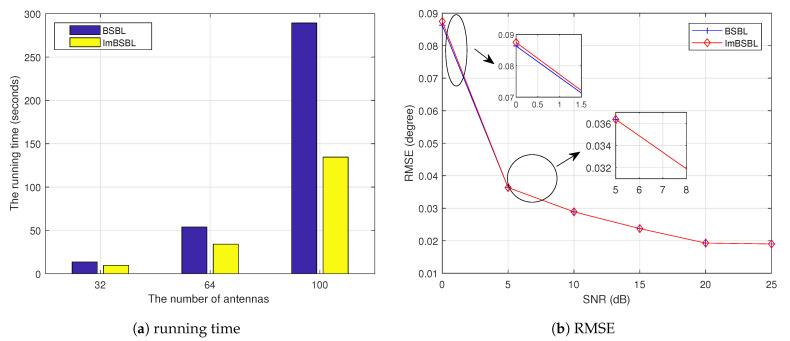
The comparison results of the BSBL algorithm and the proposed ImBSBL algorithm.

The comparison results regarding the running time and root mean squared error (RMSE) performance of the BSBL and the proposed ImBSBL algorithms are presented in Figure 5. As shown in Figure 5, the running times of both algorithms increase substantially with the number of antennas. However, since the parameters related to the convergence speed are optimized, the proposed ImBSBL algorithm has a faster convergence speed, especially when the antenna number is large. Moreover, as shown in Figure 5b, the RMSE of the proposed ImBSBL algorithm is slightly higher than that of the BSBL algorithm when the SNR is less than 5 dB. However, the two curves overlap when the SNR is higher than 5 dB. Thus, the proposed algorithm is more suitable than the BSBL algorithm for practice applications with a large number of antennas.

### 4.2. Rmse Performance Analysis

**Figure 6:**
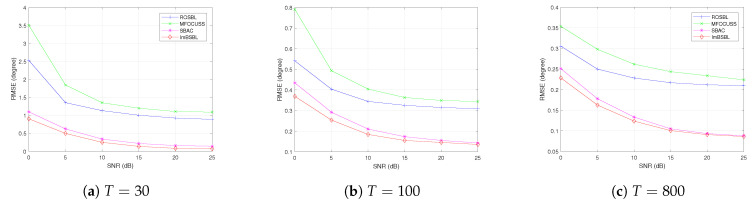
The RMSE results of DOA estimations obtained by different algorithms versus the SNR under different numbers of snapshots.

The RMSE results of different DOA estimation algorithms versus the input SNR under different snapshot are presented in Figure 6. The results are obtained under the incident angles of the signals of $-11.3^{\circ }$ and $3.7^{\circ }$ and the number of snapshots of T=30, T=100, and T=800. The SNR varies from 0 to 25 dB, while the other settings are the same as in Section 4.1 As shown in Figure 6, the RMSE performance is significantly improved with the increase in snapshot number for all algorithms; in addition, the proposed ImBSBL algorithm achieves higher estimation accuracy performance than other algorithms. In particular, the proposed algorithm could maintain excellent estimation performance even under low SNR and fewer snapshots. This can be explained by the fact that the block sparsity is used to model the signals, which can improve the reconstruction accuracy and robustness to noise. Moreover, the grid points are dynamically updated by finding the polynomial roots that can reduce the impact of the off-grid error on the estimation accuracy, coupling on estimation results. Since the ROSBL and MFOCUSS algorithms are not robust to mutual coupling, their performances are relatively poor. Although the SBAC algorithm could suppress array defects, its performance is inferior to that of the proposed algorithm because it is based on the point sparse model.

**Figure 7:**
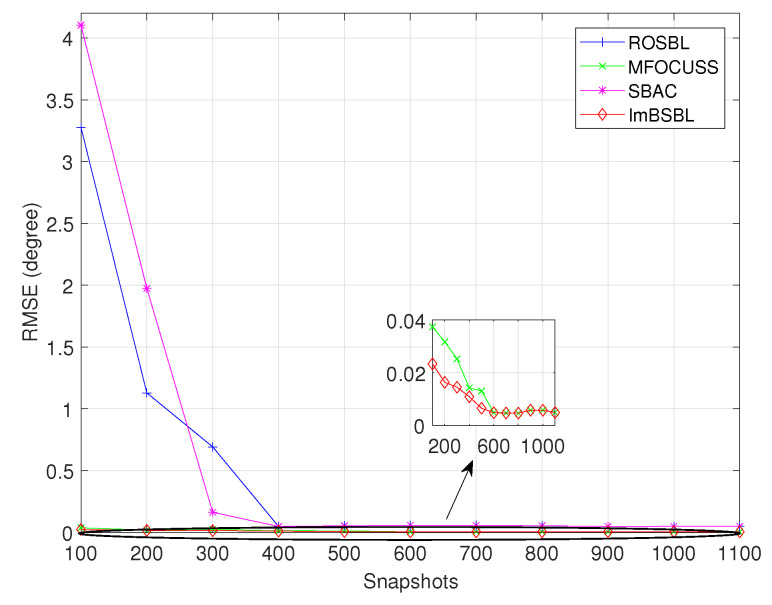
The RMSE results of DOA estimations obtained by different algorithms versus the snapshot number.

The RMSE results of the algorithms versus the snapshots number are presented in Figure 7. In this experiment, the snapshot number changes from 100 to 1100, the number of antennas is 64 and the other settings are the same as in Section 4.1. The results indicate that the average estimation accuracy of all algorithms increases monotonically with the snapshot number. When the number of snapshots is smaller than 220 and 250, the SBAC algorithm and the ROSBL algorithm fail to separate two signals, respectively, and their DOA estimation precisions are still rougher than that of the proposed ImBSBL algorithm.

**Figure 8:**
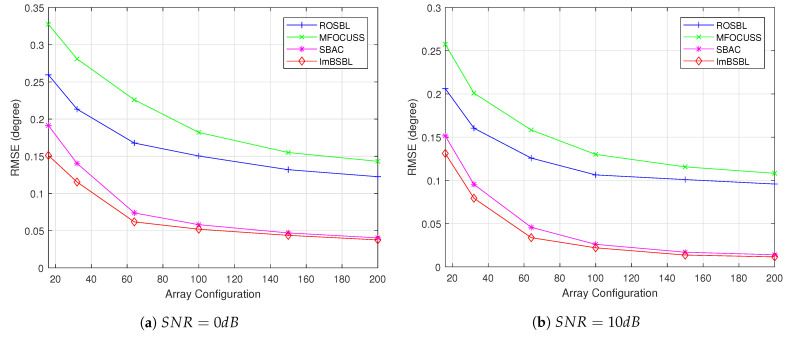
The RMSE results of DOA estimations obtained by different algorithms versus the antenna number under different SNR values.

The RMSE performance of the algorithms versus the antenna number under different SNR values are presented in Figure 8. In this experiment, the number of snapshots is 1000, the grid interval is $0.5^{\circ }$, the SNR is set to 0 dB and 10 dB, and the other settings are the same as in Section 4.1. When the SNR is fixed, the estimation performance of all methods improves significantly with the increase in antenna number. However, the SBAC algorithm and the proposed ImBSBL algorithm have smaller estimation errors than the ROSBL and MFOCUSS methods. This is because the SBAC and ImBSBL algorithms are robust to the effect of mutual coupling on the estimation results, so their performances are relatively good.

However, since the SBAC algorithm uses the point sparsity of signals for modeling, its performance is slightly worse than that of the ImBSBL algorithm, exploiting the block sparsity of signals for modeling. Thus, the proposed algorithm is suitable for large-scale MIMO systems. In addition, the estimation performances of all methods are improved when the SNR increases, but the proposed algorithm achieves better estimation performance than the other algorithms.

**Figure 9:**
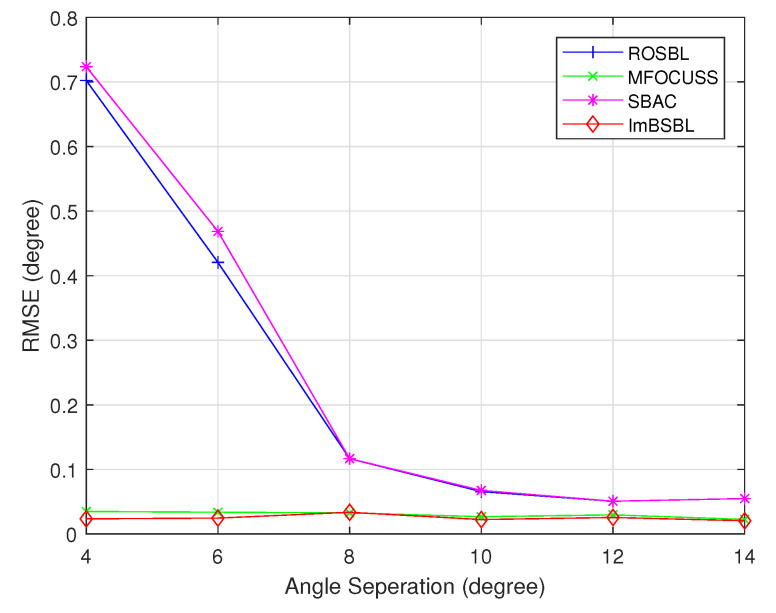
The RMSE results of DOA estimations obtained by different algorithms versus the angle separation.

The RMSE performances of the algorithms versus the angle separation are presented in Figure 9. In this experiment, DOAs of two signals are set to $\theta_{1}=-11.3^{\circ }$ and $\theta_{2}=\theta_{1}+\Delta \theta^{\circ }$, $\Delta \theta^{\circ }$ varies from $4^{\circ }$ to $14^{\circ }$, and the number of antennas is 64; the other settings are the same as those in Section 4.1. As shown in Figure 9, there is an RMSE performance gap between the SBAC and ROSBL algorithms, and HEIR estimation accuracies are much lower than those of the other two algorithms; however, the other two algorithms’ performances are significantly reduced when the angle interval is larger than $8^{\circ }$. In addition, regardless of the angle interval value, the proposed ImBSBL algorithm performs better than the MFOCUSS algorithm. This could be explained by the fact that the proposed algorithm is robust to mutual coupling and off-grid errors and explicitly use the sparsity structure of incident signals.

**Figure 10:**
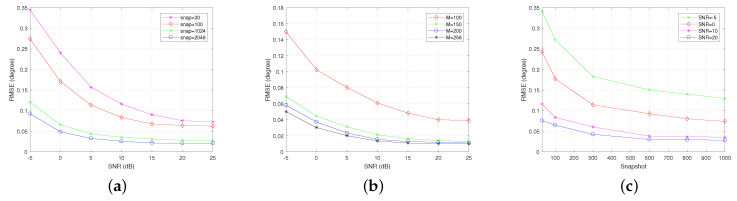
The RMSE results of the proposed algorithm. (**a**) RMSE versus SNR under different snapshots. (**b**) RMSE versus the SNR under different array element configurations. (**c**) RMSE versus the snapshots number under different SNR.

In Figure 10, the RMSE results of the proposed ImBSBL algorithm obtained by 500 independent Monte−Carlo experiments under different conditions are presented. The RMSE results versus the SNR under different snapshots numbers and array element configurations are presented in Figure 10a,b, respectively. In Figure 10c, the RMSE results versus snapshots number under different SNR are displayed. As shown in Figure 10a,c, when the SNR or snapshot number is fixed, the RMSE value of the proposed algorithm gradually decreases with the snapshots number or SNR, respectively. However, the proposed ImBSBL algorithm could maintain good estimation performance even under a small snapshot number and a low SNR. Moreover, as shown in Figure 10b, the estimation performance of the proposed algorithm could be improved by increasing the number of antennas. Consequently, the proposed algorithm achieves higher estimation accuracy under low SNR and fewer snapshots than the other algorithms; thus, it is more suitable for massive MIMO systems compared with the other algorithms.

## 5. Conclusions

This paper studies the DOA estimation problem in massive MIMO systems with unknown mutual coupling and off-grid error. Considering the mutual coupling between antennas, the studied problem can be regarded as a problem of block sparse signal reconstruction and parameter optimization. To solve this problem, this paper proposes an improved block sparse Bayesian learning algorithm to estimation the target DOAs. In the proposed algorithm, the cost function boundary is determined by exploiting the approximate approximation. Then, the parameters affecting the convergence speed are optimized to improve the convergence speed of the EM algorithm. Moreover, the effect of the off-grid error on the estimation results of the EM algorithm is considered, and dynamic updating of grid points is realized by finding the polynomial roots. Simulation results confirmed that the proposed algorithm is more robust to mutual coupling and off-grid error compared with the existing algorithms and can significantly outperform the existing DOA estimation algorithms.

## Data Availability

Not applicable.
